# Place Affect Interventions During and After the COVID-19 Pandemic

**DOI:** 10.3389/fpsyg.2021.726685

**Published:** 2021-09-14

**Authors:** Haywantee Ramkissoon

**Affiliations:** ^1^Derby Business School, College of Business, Law and Social Sciences, University of Derby, Derby, United Kingdom; ^2^Faculty of Biosciences, Fisheries and Economics, UiT, School of Business and Economics, The Arctic University of Norway, Tromsø, Norway; ^3^Johannesburg Business School, College of Business and Economics, University of Johannesburg, Johannesburg, South Africa

**Keywords:** interventions, COVID-19 pandemic, behavior change, health, mental well-being, place affect

## Abstract

The COVID-19 health and economic crisis has also brought a rise in people being unable to cope with their existing medical conditions and other issues such as domestic violence, drugs, and alcohol among others. Suicidal tendencies have been on the rise. Feelings of isolation causing emotional distress in place-confined settings have put additional pressure on the healthcare systems demanding that we find additional and complementary means of support for those in need. This is important not only in the current pandemic but also in the post-pandemic world. The goal is to collectively contribute and address the recurring calls for actions to maintain global well-being and public health. An important discussion to bring on the table is the need to promote interventions for people to cope with the pandemic and to adjust to the post-pandemic world. Promoting affective attitudes toward place can foster well-being outcomes. This has important benefits and is of relevance to governments, policymakers, and healthcare professionals in delivering better healthcare equipping people with coping mechanisms both throughout the pandemic and in the long run. However, the key challenge is how to foster these place affect attitudes meeting the changing demands in the post-pandemic world. It is in the middle of a crisis that the conversation needs to start about how to strategically plan for the recovery.

## Introduction

Pandemics such as the severe acute respiratory syndrome (SARS) in 2003, Ebola in 2014, and the Influenza A/H1N1 in 2009 and 2010 have been evidenced to impact on the mental health and well-being of the people causing financial worry, health anxiety, stress, and depression (Maunder et al., 2003; Jalloh et al., 2018; Maalouf et al., 2021). The SARS-CoV-2 pandemic is not an exception and continues to impact the world on a grand scale with the Delta variant creating a new phase in the pandemic (Kupferschmidt and Wadman, 2021). COVID-19 first emerged in December 2019 in Wuhan, China, and was declared a global health pandemic by WHO in March 2020. The main transmission of the SARS-CoV-2 starts with human-to-human contact, resulting in high infection rates across the globe (Dawood, 2020). The social and economic consequences of the SARS-CoV-2 are detrimental, and the impacts are likely to be felt over several years (Andronico et al., 2021). People keep struggling with the life-threatening virus, living in disrupted societies, countries continue to brace for the Delta variant impacts, and scientists advise we need to be concerned about the next variants (Callaway, 2021).

Most countries had borders closed for months to curb the spread of the virus. Many countries had just started reopening their borders when we were faced with the mutated virus. As a consequence, borders were closed again in several countries of the world, and the measures of precautions were strengthened. These unprecedented situations and stay-at-home orders brought adverse psychological outcomes (Tull et al., 2020). People are undergoing a lot of mental stress resulting from increased loneliness, panic attacks, financial concerns, depression, and anxiety (Luchetti et al., 2020). Public health leaders, behavioral psychologists, healthcare providers, businesses, government, and other coactors have been called to unite forces and come up with solutions to promote global public health (Ramkissoon, 2020a,b). Several countries have also adopted a multistakeholder approach (Nunkoo and Ramkissoon, 2016; Ramkissoon, 2022) to promote engagement among national and local stakeholders for collective health and well-being benefits.

The pandemic has brought negative emotions such as anger and sadness caused by sickness, loss of close ones, and bereavement (Aslam et al., 2020; Meléndez et al., 2020; Pfefferbaum and North, 2020). The COVID-19 health and economic crisis has also brought a rise in people being unable to cope with their existing medical conditions and other issues such as domestic violence, drugs, and alcohol among others. Suicidal tendencies have been on the rise (Gunnell et al., 2020; McIntyre and Lee, 2020; Pfefferbaum and North, 2020; Sher, 2020). Feelings of isolation causing emotional distress in place-confined settings have put additional pressure on the healthcare systems demanding that we find additional and complementary means of support for those in need (Ramkissoon, 2020a). This is important not only in the current pandemic but also in the post-pandemic world.

The goal is to collectively contribute and address the recurring calls for actions to maintain global well-being and public health. An important discussion to bring on the table is the need to promote interventions for people to cope with the pandemic and to adjust to the post-pandemic world. Promoting affective attitudes toward place can foster well-being outcomes (Ramkissoon et al., 2018; Townsend et al., 2018). This has important benefits and is of relevance to governments, policymakers, and healthcare professionals in delivering better healthcare, equipping people with coping mechanisms both throughout the pandemic and in the long run. Being place-confined can have negative consequences for mental health (Husky et al., 2020; Meléndez et al., 2020; Prime et al., 2020). It is important to note that place affect can also have a negative dimension when people get confined to their place as has been experienced by many across the globe during the COVID-19 pandemic. However, the key challenge is how to foster positive place affect attitudes meeting the changing demands in the post-pandemic world. It is in the middle of a crisis that the conversation needs to start about how to strategically plan for the recovery.

## Place Affect: A Subdimension of Place Attachment

Attitudes are recognized as important predictors of engaging in a specific behavior (Ajzen and Fishbein, 1977) with researchers distinguishing between cognitive and affective behaviors (Conner et al., 2013; Ramkissoon et al., 2013a,b,c). Place affect has been recognized as an attitude and important subdimension of place attachment along with the two well-recognized subdimensions of place dependence and place identity (Ramkissoon et al., 2012; Ramkissoon and Mavondo, 2015, 2017). In the last decade, place affect has been looked at across a number of disciplines, such as environmental psychology, environmental management, sociology, business, and economics. It is also attracting a lot of attention from public health scholars and in complementary, traditional, and integrative medicine.

Place affect has been defined as affective bonds individuals share with the environmental settings (Ramkissoon, 2016), fostering psychological restorativeness (Townsend et al., 2018; Ramkissoon, 2020a), and a good feeling factor. This subdimension of place attachment has been well-applied in models of decision-making. Place affect research has a focus not only on the decision or affective response to the behavior but also on the respective mood of the person (Lawton et al., 2009; Chanchaichujit et al., 2018, 2020).

There have been several messages from the health organizations to treat people who have been impacted by COVID-19 as people and not as victims. This has been generating a lot of negative sentiments, impacting their well-being. A discussion on promoting love for a place and its people during and after the pandemic is important. This study focuses on fostering affective attitudes toward a place with the aim to “undo” the negative COVID-19 emotions and behaviors.

## Place Affect and Healthy Behaviors During and After the COVID-19 Pandemic

Promoting place affect may have a positive influence on the health of the people both during and after the COVID-19 pandemic. The love of an individual for his/her place encompasses the sense of emotional bonding with his/her environmental settings. The emotional bonds formed between people and place depend on having optimal conditions in the environment, which might not necessarily be always the case for people especially when they are place-confined. The emotional bonds formed can be positive or negative depending on the relationships shared with others in a common space and the time elapsed (Okabe-Miyamoto et al., 2021). Place affect can also be about meanings that are created through the affective bonds people share with the settings of a place (Hargreaves et al., 2009; Jiang et al., 2016). These meanings can further characterize the bonding individuals develop with their environment.

### Place Affect and Well-Being Outcomes

Place affect can lead to the well-being benefits that can also be determined by the psychological interplay between the elements in a place and the emotional bonds between person and place (Völker and Kistemann, 2011; Ramkissoon, 2016). This finds support in the aesthetic-affective theory. The latter posits that the environmental settings provide the stimuli to restore the self-evaluation of an individual (e.g., one can feel more positive). In the study of Völker and Kistemann (2013), the relationship of the people with water (blue space) and clean environments have been shown to contribute to optimal health and well-being. While most people might not be living close to water, we can draw on the aesthetic-affective theory to foster positive place affect through the emotional bonding with the landscape of the house of an individual (e.g., garden) and neighborhood. Place affect has been evidenced to promote psychological restorativeness (Ramkissoon et al., 2013a,b; Ramkissoon et al., 2018) in neighborhood parks. Place affect has been associated with decreased stress levels promoting well-being (Grahn and Stigsdotter, 2010), contributing to the quality-of-life outcomes (Ramkissoon, 2020a).

People in the current pandemic and in the post-pandemic era can develop higher levels of place affect, which may have a positive influence on their everyday mood, health and well-being, and quality of life (Majeed and Ramkissoon, 2020). The love of an individual for his/her place can also stem from the “feeling-safe” factor when people are confined to their space (Ramkissoon, 2020a). People have been place confined due to the rapid spread of COVID-19. Many have had the opportunity to spend more time in the neighborhood. Further with the international closure of borders, frequent business travelers are having the luxury of spending more time in their home. This may bring a sense of deeper affection attached to their place, resulting in their psychological well-being. An example is the time that people might be able to devote to their home environment and garden settings. This in turn may make them get more bonded to their surroundings, generating high levels of place affect.

Furthermore, having experienced a more homely lifestyle, people may want to spend more time in their cities, towns, and villages and, hence, be more involved in their neighborhood after the pandemic. For instance, individuals could develop higher levels of place affect for their neighborhood parks during the pandemic and enjoy the health and well-being benefits of connecting to nature (Ramkissoon et al., 2018). Spending time in nature can also generate intentional physical exercise that is being highly recommended by health specialists and in complementary and integrative medicine (Ramkissoon, 2021). It is noted, however, that being place-confined over time can also generate conflict among people living together and impact on mental health and well-being, bringing counterproductive effects such as an increase in anxiety and stress (Husky et al., 2020; Prime et al., 2020) and breaking policy guidelines. The needs of people to connect with loved ones remain fundamental to their well-being and psychological health (Okabe-Miyamoto et al., 2021). Experiments have shown that spending more time with social connections leads to more positive emotions contributing to greater well-being outcomes (Jacques-Hamilton et al., 2019; Margolis and Lyubomirsky, 2020).

Spending time in neighborhood settings may lead to higher levels of place affect, which in turn can foster collective engagement in place-protective behaviors (Ramkissoon, 2020a,b). Evidence in the literature shows that aesthetically pleasing places can foster higher levels of place affect (Walker and Hiller, 2007; Williams, 2010), providing more opportunities for health and well-being (Mahmood et al., 2012; Coleman and Kearns, 2015). For instance, the natural beauty of peri-urban parks and waterscapes may yield psychological restorativeness (Ramkissoon et al., 2013a) and promote well-being. Researchers argue that visits to these places may foster higher levels of place affect and may encourage revisitation with further health and well-being benefits during and after the health pandemics (Majeed and Ramkissoon, 2020).

Place affect remains of particular interest to those who might not be able to travel due to the lasting impacts of the pandemic. For example, there may be people who would more carefully evaluate how and where to spend their holidays. For instance, it has been predicted by the World Tourism Organization (UNWTO) that the financial negative impacts on the SARS-CoV-2 pandemic would have fewer people traveling to remote destinations after the pandemic. With millions of people having lost their jobs, the pandemic would impact the post-COVID-19 travel behaviors. Not being able to go on holidays as before may give rise to negative emotions and cause psychological distress. It is, hence, of paramount importance to promote place affect at local destinations to maintain the physical and mental well-being of the residents. It would also be important to bring innovative approaches in urban planning. Scholars recommend incorporating more green and blue space for community health and well-being (Majeed and Ramkissoon, 2020; Salem et al., 2021).

### Place Affect, Business Recovery, and Quality of Life

COVID-19 certainly led to a set back with several natural and cultural attractions not being visited during and will probably not be visited by many in the very immediate after the pandemic. The UK government has introduced an independent review of destination management organizations to promote and strengthen the domestic tourism market and assist with a longer-term recovery from COVID-19 impacts (Visitbritain, 2021). Local places will need to transform into attractions to assist with recovery activities and restore businesses after the pandemic. Destination marketers may need to focus on place affect when redesigning their destination marketing strategies.

There has been an increase in ease of travel restrictions across Europe which is mainly due to the summer holidays. Increased mobility also brought greater contact between people mainly the youth and adolescents. The reopening of borders has enabled business recovery activities across many nations but brings significant health concerns as the delta variant is in full expansion (Burki, 2021). Some countries e.g., Australia has had the establishment of strict restrictive conditions with the prolonged international border closure to curb the spread of the virus (Adekunle et al., 2020). The prolonged pandemic continues to have policymakers debate on the public health safety measures and business recovery across several nations (Salem et al., 2021).

The unprecedented fear of COVID-19 is also likely to have some people travel less in a post-pandemic context. Several studies have reflected that the post-crisis and disaster travel behaviors of the people can be influenced by motivations (Biran et al., 2014) and risk perception (Su et al., 2015; Zheng et al., 2021).

COVID-19 also brought destructive impacts on other businesses globally (Salem et al., 2021). As a consequence, many staff have been dismissed, or having had to work from home. Organizations have also had reduced the salaries of employees. Researchers have been recommending to avoid dismissing employees during crises (Vardarlier, 2016). Losing jobs at the time of the pandemic has had severe impacts on the well-being of the people (Ramkissoon, 2020a,b; Rosemberg, 2020). The impacts will likely continue in a post-pandemic world. It remains critical for organizations during the pandemic to consider rearranging hours of work and wages for healthier societies. People suffering mental distress as a consequence of being laid off are likely to cultivate more negative place affect. This may have dire consequences in the post-pandemic world extending the suffering of the people. Notably, the survival of businesses will be governed by the global situation, yet promoting the positive psychological state of development of the employees referred to as psychological capital (Luthans et al., 2007) is being encouraged in businesses. Place affect can play a key role to keep people happy and foster coping skills in this challenging time. Promoting the positive emotions of the people can enhance their pandemic coping skills and prepare for future contingencies and better life satisfaction outcomes (Pathak and Joshi, 2020). Healthy people will be able to run healthier societies; scholars argue that positive emotions during the COVID-19 pandemic are a predictor of well-being (Schlegel et al., 2021). The health and well-being of our people remains paramount both in the current pandemic and in the post-pandemic (Ramkissoon, 2020a). This will require interventions targeted at promoting positive place affect, which may yield positive well-being outcomes in the post-pandemic era. Not only the SARS-CoV-2 pandemic is a medical and economic crisis but it can also have key declines in the subjective well-being of the people, and this calls for alternative and integrative medicine practitioners to enhance the coping skills of the people through positive affect (Ramkissoon, 2021; Zacher and Rudolph, 2021).

### Interventions Targeted at Fostering Place Affect

To combat the destructive effect of COVID-19 on jobs and the economy, there is a need for stimulus packages and interventions from governments (Higgins-Desbiolles, 2020; Ramkissoon, 2020b) for businesses to survive during and after this pandemic. There is some evidence from many countries globally showing governmental support and international aids for business recovery. Several people whose livelihoods depend on the travel and tourism industry have suffered greatly from flight cancellations, and closures of tourism businesses including the accommodation and restaurants. There have been some initiatives and government funds to continue supporting those impacted (Abate et al., 2020; Salem et al., 2021).

However, as the pandemic is still ongoing and funds are getting further utilized, there are key challenges ahead. Some governments have announced that they may stop supporting those who have lost their jobs as a consequence of the pandemic. These policy decisions have been adding to the stress of residents concerned who continue to face the uncertainty of the future. These sentiments can be further understood by the terror management theory (TMT). Terror management theory discussed how mortality salience brings along increasing anxiety and affects the overall psychological well-being of an individual (Greenberg, 1986) as they are constantly reminded of death when it is the instinctive nature of the human to survive. However, TMT also posits that anxiety can motivate people to cope with the terror, increasing the sense of the personal value of an individual (Pyszczynski et al., 2012; Hu et al., 2020). In the current pandemic, it is imperative to cope with the fear of the impacts of the pandemic to prepare for the post-pandemic new normal.

Fredrickson (1998) developed the broaden-and-build theory of positive emotions. He argued that negative emotions narrow our possibilities of fighting challenging circumstances, while positive emotions trigger thoughts and behaviors that make us better equipped to survive threats as our ancestors did. Cultivating positive emotions can work on the prolonged negative emotions (Fredrickson, 2001). Studies (Tugade and Fredrickson, 2004, 2007) evidence that positive emotions have helped people cope and bounce back from their stressful life experiences.

Building on the broaden-and-build theory, interventions need to be targeted to broaden the mindsets of the residents in times of the economic, social, and health crisis of the pandemic. By broadening their mindsets, they can be empowered to develop and/or build on personal resources that may enable them to survive through the SARS-CoV-2 pandemic and even thrive and flourish after the pandemic. A recent study shows that positive psychological states of owners and managers of small hotels during the COVID-19 pandemic promotes the recovery of business (Pathak and Joshi, 2020). Psychological empowerment can promote one's sense of attachment for his/her place (Aleshinloye et al., 2021). Promoting place affect is of paramount importance in this study to transform the mindsets and negative emotional states of the people and to transform societies equipping them to better cope with the prolonged effects of the pandemic. This may require that organizations invest more in building resilience with a focus on positive psychological states (Nilakant et al., 2016) of senior managers, mid-managers, and employees than with a focus on structural changes to better adapt to the post-pandemic world.

The literature has evidenced that individuals experiencing positive affect can better cope with crises and were found to maintain a better mental health as opposed to those who experienced more negative emotions (e.g., Fredrickson et al., 2003). In a study conducted on mental health in the aftermath of the terrorist attacks on September 11, 2001, in the United States, Fredrickson et al. (2003) found that those individuals with a higher score on the self-report measures of resilience before September 11 attacks demonstrated fewer depressive symptoms a few weeks after the attacks; their study findings show that the relationship was fully mediated by reported positive emotions.

In the current difficult circumstances of the COVID-19 global health pandemic, interventions designed at promoting place affect may assist in cultivating more positive emotions and bonds between an individual and his/her place. This would help remove the focus of the people on the negative emotions and think further on building an optimistic attitude to life. Promoting place affect can also make people more resilient, and they could be empowered psychologically, socially, and emotionally in the current challenging times and could maintain better mental, physical, and emotional health. The well-being outcomes may also be experienced after the pandemic. According to the broaden-and-build theory, people experiencing overall mental well-being will be more resilient and also more flexibly adapt their emotional states to changing life circumstances (Cohn et al., 2009; Prinzing et al., 2020). Cultivating a positive place of emotional bonding with the setting of an individual is likely to bring positive experiences. The latter is central to human health and well-being and has the potential to contribute greatly to enhance the quality of life of the people (Diener and Larsen, 1993; Myers and Diener, 1995; Ramkissoon et al., 2018; Ramkissoon, 2020a).

## Implications in a Post-COVID-19 Context and Conclusion

In preparing for the post-pandemic, we could also use the current COVID-19 as a context providing an opportunity to redesign and accommodate the needs of the aging population. Redesigning the neighborhoods with an aesthetic appeal is likely to lead to better health outcomes for the elderly. However, this will require enormous efforts in several of the heavily impacted communities on the planet. Fear has also become a social stigma for places and people heavily impacted by the pandemic (Aslam et al., 2020). We will need global collaborative efforts to design a roadmap to prioritize the place affect interventions in the poor sectors where the well-being and mental health of the people are at risk (Ramkissoon, 2021, 2022). Evidence suggests that elderly people who find their environmental surroundings to be pleasant get physically more active through engagement in activities (Mahmood et al., 2012). Elderly people are more motivated to move out of their homes to enjoy the aesthetic environment outdoors, which have important health and well-being outcomes (Walker and Hiller, 2007). They are likely to develop in-depth emotional bonds with their surroundings that can enhance their overall health and well-being. The environmental conditions of a place also represent the values that need to be shared with the present and passed on to the future generations. Promoting place affect, hence, has important benefits in the immediate and long term. These values also promote affective ties to a place and assist in personal development through positive affect. Kellert (2005) provides the example of coastal places bringing in affective, aesthetic, and spiritual opportunities providing restorative health benefits. These align with the claims that the place affect is important for the health and well-being of the people (Ramkissoon, 2020a) and in turn can enhance the overall quality of life (Ramkissoon, 2020b). Promoting place affect is recommended to urban planners, policymakers, healthcare leaders, and other coactors to promote global health and well-being and align with the UN Sustainable Development Goals. Recognizing the important role of promoting place affect in the place of belonging of the people, the author noted that promoting place affect interventions remains one of the proposed interventions in preparing for a post-pandemic world; the pandemic is multifaceted and needs to be addressed from multiple dimensions (Páez and Pérez, 2020).

## Author Contributions

The author confirms being the sole contributor of this work and has approved it for publication.

## Funding

UiT, The Arctic University of Norway has supported the open access charge.

## Conflict of Interest

The author declares that the research was conducted in the absence of any commercial or financial relationships that could be construed as a potential conflict of interest.

## Publisher's Note

All claims expressed in this article are solely those of the authors and do not necessarily represent those of their affiliated organizations, or those of the publisher, the editors and the reviewers. Any product that may be evaluated in this article, or claim that may be made by its manufacturer, is not guaranteed or endorsed by the publisher.
